# Imaging retinal microvascular manifestations of carotid artery disease in older adults: from diagnosis of ocular complications to understanding microvascular contributions to cognitive impairment

**DOI:** 10.1007/s11357-021-00392-4

**Published:** 2021-06-08

**Authors:** Lilla István, Cecilia Czakó, Ágnes Élő, Zsuzsanna Mihály, Péter Sótonyi, Andrea Varga, Zoltán Ungvári, Anna Csiszár, Andriy Yabluchanskiy, Shannon Conley, Tamás Csipő, Ágnes Lipecz, Illés Kovács, Zoltán Zsolt Nagy

**Affiliations:** 1 Department of Ophthalmology, Semmelweis University, 39 Mária Street, 1085 Budapest, Hungary; 2 Department of Vascular & Endovascular Surgery, Semmelweis University, Budapest, Hungary; 3 Department of Biochemistry and Molecular Biology, Vascular Cognitive Impairment and Neurodegeneration Program, Center for Geroscience and Healthy Brain Aging, University of Oklahoma Health Sciences Center, Oklahoma City, OK USA; 4 International Training Program in Geroscience, Doctoral School of Basic and Translational Medicine/Department of Public Health, Semmelweis University, Budapest, Hungary; 5 Department of Health Promotion Sciences, College of Public Health, University of Oklahoma Health Sciences Center, Oklahoma City, OK USA; 6 International Training Program in Geroscience, Theoretical Medicine Doctoral School/Departments of Medical Physics and Informatics & Cell Biology and Molecular Medicine, University of Szeged, Szeged, Hungary; 7 International Training Program in Geroscience, Doctoral School of Basic and Translational Medicine/Department of Translational Medicine, Semmelweis University, Budapest, Hungary; 8 Department of Cell Biology, University of Oklahoma Health Sciences Center, Oklahoma City, OK USA; 9 Department of Ophthalmology, Josa Andras Hospital, Nyiregyhaza, Hungary; 10 Department of Ophthalmology, Weill Cornell Medical College, New York City, NY USA; 11 Department of Clinical Ophtalmology, Faculty of Health Sciences, Semmelweis University, Budapest, Hungary

**Keywords:** Retinal biomarkers, Carotid artery stenosis, Retinal imaging, OCT angiography, Vascular dementia, VCID

## Abstract

Carotid artery stenosis (CAS) is a consequence of systemic atherosclerotic disease affecting the aging populations of the Western world. CAS is frequently associated with cognitive impairment. However, the mechanisms contributing to the development of vascular cognitive impairment (VCI) associated with CAS are multifaceted and not fully understood. In addition to embolization and decreased blood flow due to the atherosclerotic lesion in the carotid artery, microcirculatory dysfunction in the cerebral circulation also plays a critical role in CAS-related VCI. To better understand the microvascular contributions to cognitive decline associated with CAS and evaluate microvascular protective effects of therapeutic interventions, it is essential to examine the structural and functional changes of the microvessels in the central nervous system (CNS). However, there are some limitations of in vivo brain vascular imaging modalities. The retinal microvasculature provides a unique opportunity to study pathogenesis of cerebral small vessel disease and VCI, because the cerebral circulation and the retinal circulation share similar anatomy, physiology and embryology. Similar microvascular pathologies may manifest in the brain and the retina, thus ocular examination can be used as a noninvasive screening tool to investigate pathological changes in the CNS associated with CAS. In this review, ocular signs of CAS and the retinal manifestations of CAS-associated microvascular dysfunction are discussed. The advantages and limitation of methods that are capable of imaging the ocular circulation (including funduscopy, fluorescein angiography, Doppler sonography, optical coherence tomography [OCT] and optical coherence tomography angiography [OCTA]) are discussed. The potential use of dynamic retinal vessel analysis (DVA), which allows for direct visualization of neurovascular coupling responses in the CNS, for understanding microvascular contributions to cognitive decline in CAS patients is also considered.

## Introduction

Aging is a major risk factor for cardiovascular and cerebrovascular diseases, and advanced age is associated with increased prevalence and worsened prognosis of cardiovascular and cerebrovascular diseases. Carotid artery stenosis (CAS) is an important, potentially life-threatening consequence of systemic atherosclerotic disease in the aging population. A meta-analysis of four population-based studies (Malmö Diet and Cancer Study, Tromsø study, Carotid Atherosclerosis Progression Study, and Cardiovascular Health Study) showed that prevalence of moderate (≥ 50% stenosis) asymptomatic CAS ranges from 0.2% in men aged < 50 years to 7.5% in men aged ≥ 80 years[1]. The prevalence of severe (≥ 70% stenosis) asymptomatic CAS ranges from 0.1% in men aged < 50 years to 3.1% in men aged ≥ 80[1]. While the prevalence of moderate and severe CAS in women is somewhat lower, the dramatic, age-related increase in prevalence is still present: at 5.0% in women ≥ 80 and 0.9% in women < 50 years of age [1–3]. CAS affects a significant percentage of the aging population, and is responsible for 10–20% of the ischemic strokes, which are the second most common cause of death worldwide [4, 5]. Established risk factors promoting progressive atherosclerosis in the peripheral circulation can also potentially exacerbate atherogenesis in the carotid arteries, resulting in CAS. Accordingly, the most important risk factors of CAS include age over 50 years, smoking, hyperlipidemia, diabetes, obesity, occurrence of a cardiovascular event in a family member younger than 60 years, peripheral arterial disease, coronary disease, stroke or transient ischemic attack (TIA) in the medical history [2].

Patients with carotid artery disease frequently exhibit cognitive impairment [6–11]. The mechanisms contributing to the development of vascular cognitive impairment (VCI) associated with carotid artery disease are multifaceted. Severe CAS can limit blood flow to the brain. Hemodynamic insufficiency resulting in ischemic brain damage may occur when pathological conditions that compromise regulation of cerebral perfusion (e.g., orthostasis, hypotension, volume depletion, or cardiac failure) are superimposed on cerebral hypoperfusion caused by CAS. Emboli derived from the atherosclerotic lesions can occlude cerebral vessels, causing ischemic strokes in the supplied brain regions. In addition, it is becoming increasingly evident that CAS is also associated with microcirculatory dysfunction in the cerebral circulation [12–15]. The smaller cerebral arterioles are the primary determinants of cerebral vascular resistance and thereby regional cerebral blood flow. Consequently, pathological changes in the structure and function of these smaller vessels can have a significant impact on oxygen and nutrient supply to the neuronal tissue. Atherosclerotic vascular diseases, including CAS, are chronic inflammatory diseases of the larger arteries. Yet, systemic pathological processes underlying the development of CAS also affect the cerebral resistance vessels. There is growing evidence that consequences of CAS extend into the cerebral microcirculation, contributing to the development of small vessel disease and promoting micro-infarcts[6, 16], white matter damage[17–20] and lacunar infarcts [21, 22] and thereby exacerbating the pathogenesis of VCI. Additionally, pathological processes underlying the development of CAS also result in pathological remodeling of the microcirculatory network [23–28]. Microvascular rarefaction, including a decline in capillarization, is also considered a critical factor contributing to impaired brain perfusion and the development of VCI [29].

Additionally, an important element in our current understanding of the pathogenesis of VCI is the role played by endothelial cells in the regulation of cerebral blood flow [29]. The cerebromicrovascular endothelium plays a key role in modulation of vascular tone and microcirculatory network resistance through the release of a variety of vasoactive mediators, including NO and prostaglandins [29, 30]. Healthy endothelial function is also needed for moment-to-moment adjustment of regional blood flow to the increased energy requirements of active neurons via neurovascular coupling [29, 31–37]. There is direct evidence that impaired endothelium-mediated vasodilation in the cerebral microcirculation is causally involved in the genesis of cognitive impairment [31, 32, 38]. Importantly, the pathological conditions associated with the premature development of CAS (e.g., aging, hypercholesterolaemia, untreated hypertension, smoking, and diabetes mellitus) have been associated with significant endothelial dysfunction, which results in dysregulation of cerebral blood flow [29].

Understanding microvascular manifestations of CAS is essential for preventing cognitive decline and reducing cerebrovascular mortality in this high-risk population. In this review, the effect of CAS and the pathological processes underlying CAS on the functional and structural integrity of the CNS microcirculation is considered. There are useful methods available to study the brain microcirculation and regulation of cerebral blood flow in humans, ranging from functional magnetic resonance imaging (fMRI)-based approaches to functional Near-Infrared Spectroscopy (fNIRS). Because many of these methods have important limitations (including low resolution, high costs, long measurement time, time-consuming analyses, equipment availability, invasiveness, and side effects of contrast agents), there is an urgent need for sensitive, non-invasive methods that can be used both in large-scale cross-sectional studies and in longitudinal studies to assess treatment efficiency.

We present an argument for the view that the direct imaging of retinal microvessels can be used as a proxy measure to study pathological processes underlying cerebral small vessel disease and VCI. The retina is anatomically an extension of the brain (during development the retina is formed as an outgrowth of the embryonic diencephalon) and is considered part of the CNS. The retinal and brain microcirculations share many anatomical and physiological features and the existing evidence strongly suggest that microcirculatory functional and structural alterations in the retina correlate closely with those in the brain. The potential use of novel imaging methods to assess the combined functional and structural impairment of the retinal microcirculation in CAS patients is discussed.

## Carotid artery disease

### Clinical characteristics

It is important to distinguish the asymptomatic and symptomatic CAS cases as they are associated with differential risk of stroke. According to the Asymptomatic Carotid Surgery Trial, in asymptomatic cases with a degree of the stenosis between 60 and 99%, the annual stroke risk is 0.7–1.1%/year [39]. Cases are considered symptomatic when a stenosis higher than 50% is present and the patient had symptoms indicating stroke or TIA within the last 6 months ipsilateral to the lesion [5]. Such symptoms are contralateral monoparesis, hemiparesis or paresthesia, dysarthria, dysphagia, aphasia, ipsilateral amaurosis fugax or blindness. The risk of a new stroke exponentially increases with the elapsed time since the presentation of the last symptom. Within 2 days of a TIA, the probability of stroke is 6.7% (this level of risk is approximately equivalent to 10 years of accumulated risk for asymptomatic patients), and the stroke rate within two weeks is 10% [40]. Therefore, secondary stroke prevention by carotid artery reconstruction has a more prominent effect in stroke risk reduction when compared to the asymptomatic patients. For this reason, guidelines recommend reconstruction within 2 weeks of TIA or minor stroke, provided there are no contraindications to surgery [41]. The prevalence of stroke is significantly lower in patients who undergo carotid endarterectomy compared to those receiving pharmacological therapy [42, 43].

#### Molecular mechanisms underlying carotid artery stenosis (CAS)

**Fig. 1:**
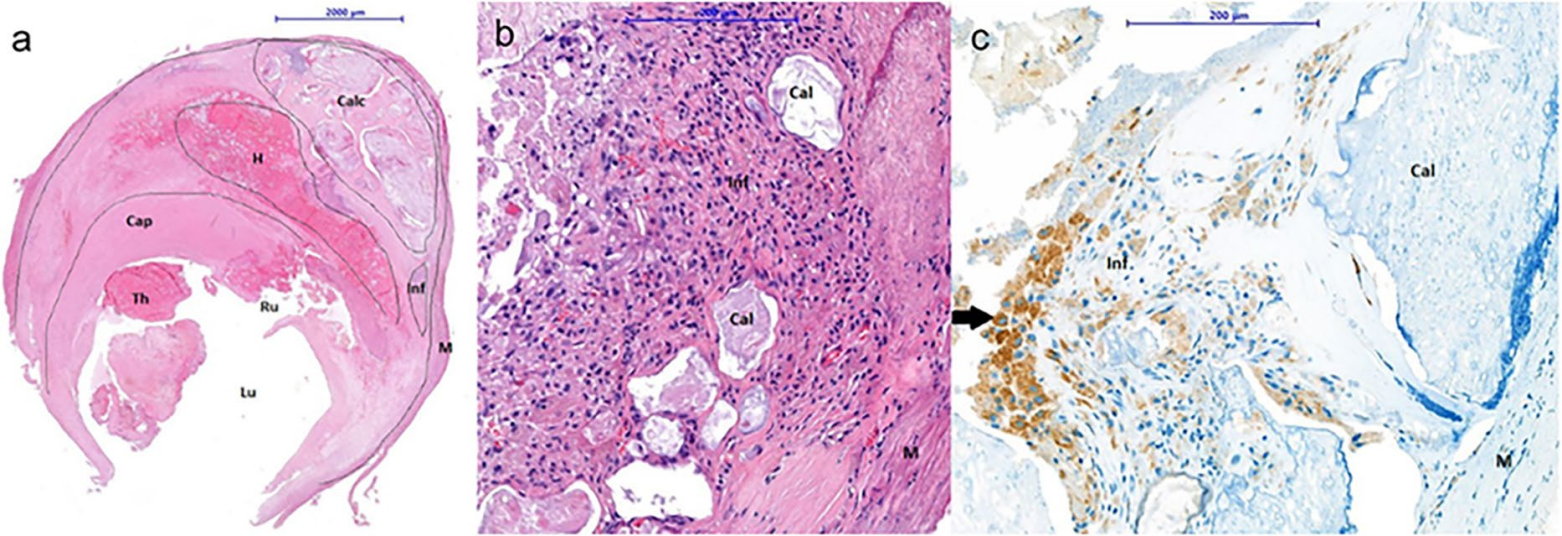
Representative cross-section of an unstable carotid plaque removed during carotid endarterectomy stained by HE. The plaque is composed of a large amount of fibrotic tissue in the plaque and the cap of the plaque. Grade 4 plaques are characterized by a rupture (Rup) in the fibrous cap of the plaque (Cap), presence of a thrombus (Thr) and intraplaque hemorrhage (Hem); inflammation (Inf); calcification (Cal); medial smooth muscle cells (M); lumen (Lum) (**a**). A representative magnified image of inflammatory cells plaque tissue stained by HE (**b**). An image with CD68 positive macrophage staining. Arrows points to positively stained cells in plaque tissue. The high number of CD68 positive macrophages and foam cells in the plaques are histological features of the unstable plaques (**c**) [51].

Atherosclerotic lesions in the carotid bifurcation can lead to cerebral embolization by rupture of the fibrous cap, which can expose intensely thrombogenic materials to blood elements [44]. The molecular changes in the carotid vessel wall during plaque formation have been studied extensively before, the data showed diversified and complex systematic changes in carotid plaques. Previous microarray analysis studies compared the plaque of symptomatic and asymptomatic patients. All of them concluded that symptomatic plaques are molecularly and biochemically (immune, inflammatory, cell signaling and protein defining processes) altered from the asymptomatic plaques [45–48]. However, functional analysis of these candidate genes has not been performed, thus any of the genes’ differential expression has not proven to be a precursor to symptom onset. In a recent study, RNA expression, proteomic and IHC analysis found that *ABCA1* CD44, KLF2, PLIN2, ferritin, ACTB, CAIX, and ENO1 protein *levels* were significantly higher in ruptured plaques than in non-ruptured plaques. Bioinformatic analysis suggested that only ABCA1 has a preventive role in carotid plaque rupture and atherosclerosis progression during the inflammatory reaction [49]. There are many IHC markers (CD3, CD68, CD31, CD34, MMP9) expression in special localization in the plaque, which were suspected to be useful to identify the high-risk plaques [50–52]. (In Fig. 1, CD68 macrophage and foam cells are presented in high quantity, which is a typical, unstable plaque histopathological feature.) Beyond the previous findings, a microarray analysis of carotid plaques suggests, there are regional differences in protein expression along the long axis of the carotid plaque. It was suggested for further investigations to focus on the rupture-prone postbifurcation segment’s gene expression [53]. Nonetheless, not only the single genes and non-validated proteins but the unlocated ones without context could lead to false assumptions.

Proteomics and other detailed cellular and molecular investigations are needed to clarify the molecular characteristics of symptomatic vs asymptomatic plaques and unstable or ruptured plaques vs stabile plaques, and to suit them to the high-risk carotid plaque imaging features, which were classified by AHA (American Heart Association) [54]. Six features are linked to carotid plaque vulnerability: intraplaque hemorrhage, lipid-rich necrotic core and fibrous cap, plaque inflammation and intraplaque neovascularisation, plaque thickness, surface morphology, and plaque volume [55]. The problematic impact of calcification on plaque instability is also a widely researched topic for studies based on the comparison of histopathological and imaging plaque feature studies. The latest findings show that macrocalcification leads to plaque stability, while microcalcification is more likely to be associated with plaque rupture [56].

The comparison of the microarray studies’ target genes and proteins to imaging and histopathological features are the main goal in translational researches. Clinical scoring system will be based on these findings [51]. Furthermore, its automatic evaluation needs further investigation and evolution in machine learning and AI development. There is also a great demand for studies on circulating biomarkers based on the recognition of the exact molecular mechanisms of carotid plaque instability, which could provide additional help to predict the risk of atherothrombotic stroke in daily clinical practice [44].

### Current protocols of carotid artery stenosis (CAS) management

#### Diagnosis

**Fig. 2:**
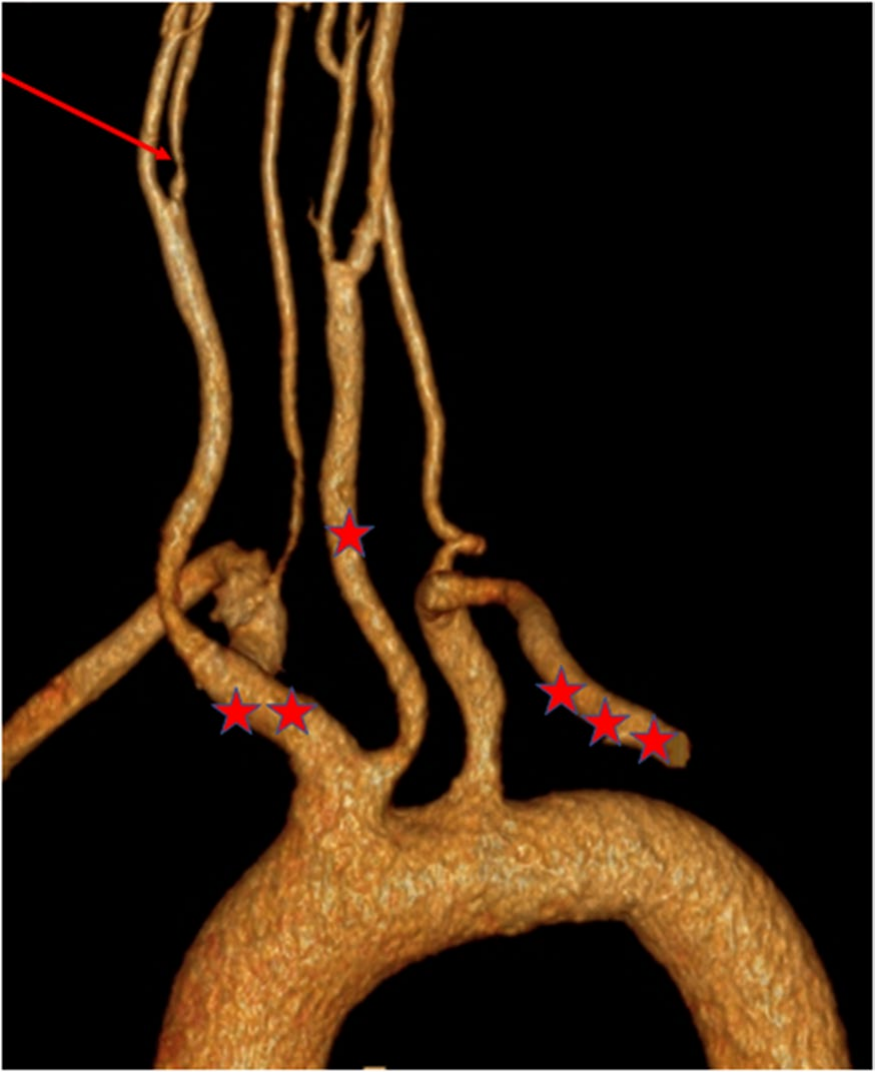
The supra aortic branches of the aorta are shown in the 3D reconstruction of CT angiography. A red asterisk signs the brachiocephalic trunk; it branches to the right common carotid artery and the subclavian artery. The red arrow shows the significant stenosis of the right internal carotid artery distal from the carotid bifurcation. Two red asterisks sign the left common carotid artery. Three red asterisks sign the left subclavian artery second part distal form the origin of the left vertebral artery

In current practice, the preferred method for diagnosis and evaluation of the severity of carotid artery stenosis is Duplex ultrasound. This fast, cheap and highly accurate method gives not only morphological information but also makes it possible to evaluate the extent of the stenosis by measuring the different flow velocities. Although Doppler ultrasound (DUS) can be a reliable examination method if performed by an experienced examiner, previous studies have shown that several human factors result in an increase in error and variability [57, 58]. CT- and MR (magnetic resonance) angiography are more objective methods than DUS. The latter imaging methods are mostly used in reconstruction planning thanks to their ability to provide suitable images of the blood vessels from the aortic arch to the intracranial vessels as well as the ischemic lesions of the skull (Fig. 2). The degree of stenosis in CTA and MRA is calculated according to the criteria developed by NASCET [43], where the residual lumen at the most stenotic portion of the proximal ICA is compared to the distal normal post-stenotic ICA diameter.

Currently, conventional digital subtraction angiography (DSA) is not the first-line method for the assessment of carotid stenosis. It should not be performed in patients considered for revascularization, unless there are significant discrepancies on non-invasive imaging (E.g., DUS, CTA, or MRA).

According to the ESVS guideline [41], when CEA is being considered, it is recommended that DUS stenosis estimation is corroborated by CTA or MRA. If CAS is being considered, it is also recommended that any DUS study be followed by CTA or MRA to provide additional information on the aortic arch, as well as the extra- and intracranial circulation.

Although the aforementioned imaging methods are effective in clinical practice and enable the evaluation of the degree of stenosis, the effect on the downstream microcirculation of the CNS cannot be assessed.

#### Treatment

In asymptomatic cases when the extent of the stenosis is less than 70%, pharmacological treatment is recommended (daily administration of thrombocyte aggregation inhibitors and statins) as well as lowering the modifiable risk factors [41]. Pharmacological treatment is also the preferred alternative in cases where the risk of an intervention is considered too high compared to their potential preventive value. Regarding the surgical treatment options, the first line of treatment is endarterectomy since this procedure has the lowest perioperative stroke rate [59, 60]. The surgery can be performed under general or locoregional anesthesia and consists of the removal of the calcified endothelium and median layer of the vessel. The two variations to perform the procedure are conventional endarterectomy when patch angioplasty is used to close the wound and eversion endarterectomy during which the intima and media layer is everted. The advantage of the latter is that this approach is free of non-autologous materials which means fewer infections and patch degeneration in addition to lower postoperative embolization and restenosis rates. Carotid artery angioplasty and stent implantation followed by lifelong medical therapy is the preferred treatment in cases when the perioperative risk is considered too high, there is previous cervical operation or irradiation in the medical history of the patient, in cases of restenosis and contralateral recurrent nerve palsy [61]. A lifelong administration of statins and thrombocyte aggregation inhibitors is essential following surgery along with regularly scheduled ultrasound examinations [41].

### Cognitive decline associated with carotid artery stenosis (CAS)

Patients with CAS frequently exhibit cognitive impairment [6–10]. The mechanisms contributing to the development of VCI associated with carotid artery disease are multifaceted. Cardiovascular risk factors (i.e., hypertension, diabetes, dyslipidemia, and smoking) are also risk factors for stroke, dementia, and CAS [8]. Presence of ischemic brain injury with symptomatic CAS can directly contribute to cognitive impairment, however, several studies also suggested that asymptomatic CAS may act as an independent risk factor for cognitive impairment [9]. The Cardiovascular Health Study evaluated an elderly population (aged ≥ 65 years), without a history of stroke or TIA [62]. The study found that high-grade (> 75%) CAS was associated with cognitive impairment and cognitive decline during the five-year follow-up period, and importantly, this association was independent of MRI-detectable signs of cerebral ischemia. Support for these findings came from the Tromsø study, which investigated degree of CAS, brain morphology, and cognitive function. Participants in the CAS group had a CAS degree greater than 35%, and the study demonstrated that CAS patients performed worse on several subsets of cognitive tests compared to controls, and this association was again independent of MRI-detectable brain lesions [10]. Furthermore, a significant association was observed between the impairment of cognitive performance and the degree of CAS [10].

### The effect of carotid artery stenosis on cerebral blood flow

Significant stenosis or occlusion of the carotid artery results in cerebral hypoperfusion if compensatory mechanisms are exhausted [63]. Cerebrovascular dilation capacity is also compromised in CAS patients with severe stenosis [64]. Interestingly, Sundt et al. reported that surgical treatment of CAS via carotid endarterectomy only resulted in an increase in cerebral blood flow if the degree of stenosis was greater than 90% [65].

Energy demand in the brain varies both spatially and temporally with changes in neuronal activity, thus requiring prompt adjustments of regional cerebral blood flow (“functional hyperemia”) in a highly regulated fashion to maintain cellular homeostasis and function [29, 36, 66, 67]. This adjustment is accomplished through a homeostatic regulatory mechanism termed neurovascular coupling [29, 36, 37, 68–70]. The cellular mechanisms underlying neurovasuclar coupling are complex, but include activation of astrocytes by firing neurons, followed by the release of vasodilator gliotrasmitters which promote localized microvascular dilation via, in part, via an endothelium-dependent pathway [29, 31, 32, 34, 37, 68, 71]. Inadequate augmentation of blood flow during neuronal activation leads to a mismatch between supply and demand of oxygen and metabolic substrates and impairs wash-out of harmful metabolites in functioning cerebral tissue [72]. There is convincing evidence that impaired neurovascular coupling per se can compromise neuronal and cerebral function [73–79]. Experimental studies provide direct support for a causal role of neurovascular uncoupling in the genesis of cognitive impairment. For example, pharmacologically induced neurovascular dysfunction was shown to impair spatial and recognition memory in mouse models [38]. Further, there are also studies showing that rescue of neurovascular coupling in animal models of VCI results in significant improvement of cognition [31, 32]. In light of these data it is significant that CAS is associated with impaired neurovascular coupling responses in the brain [80].

Cerebral autoregulation is a critical homeostatic mechanism that maintains relatively stable cerebral blood flow despite changes in the cerebral perfusion pressure. A network model study of the cerebral autoregulation and collateral flow in circle of Willis found that collateral flow alone is unable to ensure adequate blood flow after major vessel occlusion [81]. However, results of the same study suggested that status of the autoregulatory ability of the circle of Willis network is of major importance when assessing collateral blood supply. In fact, the autoregulatory capacity of the circle of Willis can be a good predictor of ischemic disease risk and cerebrovascular intervention outcomes [82]. In cases of obstructive carotid artery disease associated with chronic impairment of cerebral blood flow, detection of impaired cerebral autoregulation might help to identify patients at risk from stroke similar to what has previously been shown for patients with impaired cerebrovascular reserve capacity [81–83].

**Fig. 3:**
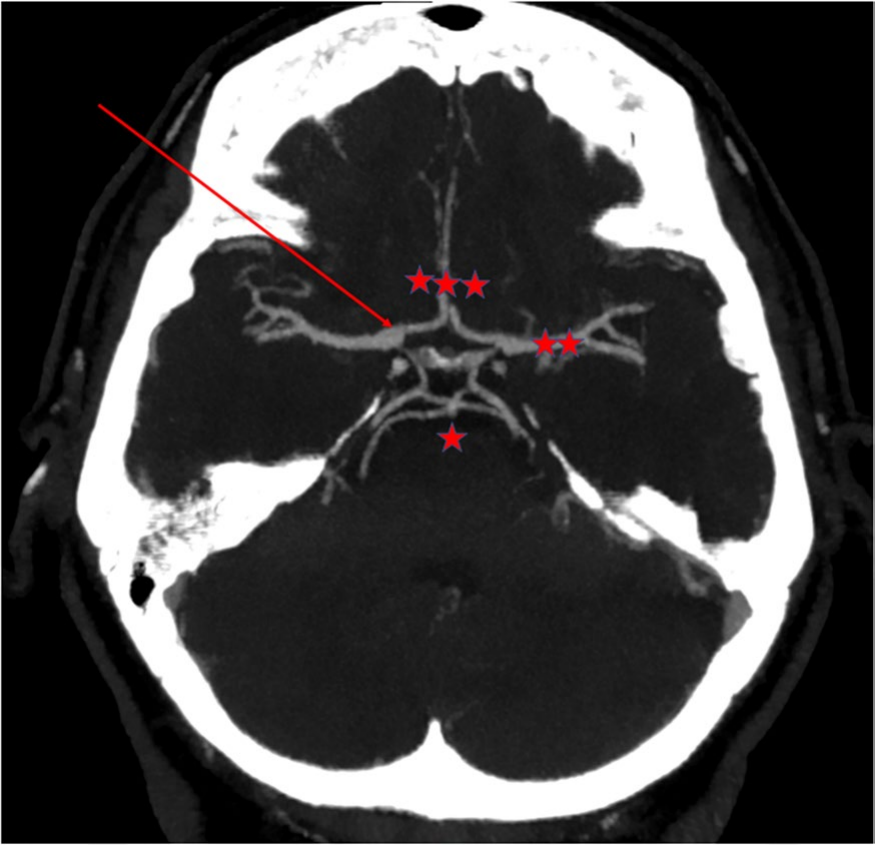
The CT angiography shows the circle of Willis (CW), which is the primary collateral system of the patients with steno-occlusive disease of the carotid. The red arrow signs the right internal carotid artery. It branches the medial cerebral artery (two red asterisks), the anterior cerebral arteries and the anterior communicant artery (three red asterisks), which constitute the anterior circle of CW. The one red asterisk signs the basilar artery, which branches the posterior cerebral artery and the posterior communicant arteries creating the posterior circle of CW

Collateral recruitment can partially compensate cerebral perfusion in patients with steno-occlusive disease of the carotid arteries [63]. The primary collaterals within the circle of Willis (CW; Fig. 3) are the main collaterals responsible for maintaining sufficient perfusion of the affected vascular territory in cases of severe internal carotid artery stenosis, while the collaterals from the leptomeningeal and external carotid arteries play a larger role in cases of poor hemodynamic status [84]. Thus, the anatomical characteristics of the circle of Willis significantly affect the outcome of CAS. The prevalence of less common circle of Willis structural variants is significantly higher in patients who underwent carotid artery reconstruction compared to controls [85]. In addition, the absence of collateral segments (or the presence of dysfunctional segments) can be associated with higher risk of cognitive impairment, TIA, and ischemic stroke in symptomatic CAS patients and it can increase the risk of neurological events after carotid artery reconstruction without shunting [86, 87].

Importantly, functional hyperemia/neurovascular coupling and vascular autoregulation also occur in the retina. Studies using animal models have demonstrated that retinal neurovascular coupling shares many cellular mechanisms with neurovascular coupling in the brain, including a role for glia, neurons, pericytes, and endothelial cells [88, 89]. In addition, newer imaging modalities (further discussed below) have made it much easier to measure retinal neurovascular coupling in humans, and impaired functional hyperemia in the retina is thought to play a role in various conditions including aging, glaucoma, diabetic retinopathy, and other neurodegenerative diseases [90–92]. Similarly, pressure-dependent changes in retinal blood flow (autoregulation) have been recorded in animal models such as cats [93]. Retinal autoregulation also occurs in humans, where it is critical to maintain adequate retinal perfusion in the face of systemic hypotension or increased intraocular pressure (for example in glaucoma) [94–96]. Until recently, imaging limitations made retinal autoregulation hard to measure well, but newer approaches such as OCT angiography have helped overcome this limitation [95, 96]. These physiological similarities between brain and retina vascular regulation provide support for the use of the retina as a proxy for evaluation of cerebrovascular microcirculatory defects associated with cardiovascular disease.

### Cerebral small vessel disease associated with CAS

Cerebral MRI frequently reveals signs of cerebral small vessel disease in the form of white matter lesions and lacunar infarcts in CAS patients [21, 97, 98]. Cerebral small vessel disease has been causally linked to the genesis of dementia [99, 100]. Despite its importance, the pathological status of cerebral small vessels is difficult to assess in vivo.

## Imaging retinal structures and the retinal microvasculature in CAS patients

### Understanding microvascular pathologies associated with CAS by examining the retinal microvasculature

The retinal microvasculature provides unique opportunities to study pathogenesis of cerebral small vessel disease, because the cerebral circulation and the retinal circulation share similar anatomy, physiology and embryology. The direct visualization of retinal vessels using the methods detailed below provides a unique opportunity to study retinal microvascular health as a proxy for brain microvascular health in patients with CAS.

The blood supply of the retina is provided by the retinal and choroidal vasculature. Both are derived from the ophthalmic artery, the first branch of the internal carotid artery. As a result, changes in blood flow parameters in the internal carotid artery may result in ophthalmic complications. Pathophysiological changes affecting the CNS and the cerebral microcirculation can also affect the retina and retinal microcirculation through shared cellular, molecular, thromboembolic and hemodynamic mechanisms. Further, the retina may also serve as an area of the CNS where pathological changes in tissue structure can be directly imaged for diagnostic purposes. The retina shares developmental origins with the brain as they derive from the same pluripotent neuroectodermal cells of the diencephalon. Thus, the retina can be viewed as an extension of the central nervous system [101]. Just like the brain, the retina is a highly metabolically active tissue and is among those with the highest oxygen demand in the body. There are also several similarities between the retinal and the cerebral microcirculation such as having end arteries without anastomoses, playing a barrier and autoregulatory role, and being relatively low-flow/high-oxygen-extraction systems [102]. In common with the brain, the retina has little local oxygen and energy reserves; therefore, it also relies on functional hyperemia to meet the need of activated neurons. Dynamic adjustment of regional blood flow in areas of neuronal activation occurs through neurovascular coupling, and the retina is the only area of the CNS where neurovascular coupling can be directly assessed with non-invasive methods [103].

Several established methods exist to image the ocular circulation (e.g., funduscopy, fluorescein angiography); however, these methods do not allow quantitative measurement of the blood supply. In the case of CAS, the existence of collateral blood supply means that imaging retinal anatomy may not detect altered blood supply of the retina. Doppler sonography of ocular arteries provides more information about the blood supply than simple angiography; however, it is limited to large ophthalmic arteries and is therefore less informative for study of the retinal microcirculation. Some newer methods are capable of providing better functional measurements. Optical coherence tomography (OCT) provides high resolution, cross-sectional morphological imaging of the retina and the choroid, while optical coherence tomography angiography (OCTA) is able to image the retinal vasculature by detecting the movement of red blood cells in consecutive scans. In addition, a novel method, dynamic retinal vessel analysis (DVA), allows investigation of the dynamic regulation of blood supply in the retina in response to neuronal activation by flickering light [104, 105].

### Fluorescein angiography (FA)

For several decades, fluorescein angiography (FA) has been the gold standard procedure for visualizing the retinal circulation. For this dye-based imaging technique, fluorescein dye is injected into a peripheral vein in the arm or hand. Thereafter the retina is illuminated with blue light. The dye emits a fluorescent green light which is recorded to image the retinal vasculature. FA enables the dynamic evaluation of contrast movement in real time. It provides information on the retinal vascular perfusion and the integrity of the inner blood-retinal barrier. Pictures can be obtained of the peripheral retina as well as the posterior pole of the eye. A notable disadvantage of FA is the inability of the method to provide quantitative information about the retinal blood supply.

### Doppler ultrasonography of the ophthalmic artery

Doppler imaging has been used for a long time in imaging of orbital structures. Color Doppler imaging provides real-time information on the blood flow in a color-coded format. The method is suitable for examination of the ophthalmic artery, the central retinal artery and the posterior ciliary artery [106]. However, it is not capable of providing functional information on the retinal microcirculation, so is of limited utility for assessing microcirculatory complications of disease.

### Optical coherence tomography (OCT)

Optical coherence tomography (OCT) is a rapid, non-invasive and non-contact imaging technique which allows in vivo visualization of retinal structures, the optic nerve head, and the retinal nerve fiber layer (RNFL) using low coherence interferometry [107]. Since its introduction in 1991 it became widely utilized in diagnostics and longitudinal follow-up of retinal diseases such as diabetic retinopathy, age-related macular degeneration, and glaucoma (where RNFL thickness is commonly affected) [108, 109]. OCT can obtain high-resolution cross-sectional images enabling clear visualization and measurement of the thickness of retinal layers. Unfortunately, the contrast between retinal vessels and the surrounding tissue is insufficient for resolving vascular structure, thus retinal OCT is not suitable for evaluating vascular outcomes.

### OCT angiography (OCTA)

A novel method for visualizing and analyzing the retinal and choroidal vasculature is optical coherence tomography angiography (OCTA) which is able to visualize the retinal and choroidal vasculature without the use of an intravenous dye. The imaging technique is based on motion contrast technology to detect the movement of the red blood cells within the vessels. It enables the accurate visualization of the microvasculature of the macular area and around the optic disc, and also provides quantitative information such as the vessel density (VD) in said areas and the size of the foveal avascular zone (FAZ). The examination is fast and easily repeatable and importantly, provides simultaneous structural and functional blood flow information. The method also has its limitations, the smallest capillaries where the velocity of blood cells is under the detectable threshold can’t be visualized and bleeding and leakage cannot be seen either. With the currently used OCT devices and software, only the central parts of the retina can be measured: a 3–8 mm square of the macular area or a 4.5–6 mm square of the peripapillary area. The most important limiting factors for OCTA are the ones affecting the image quality such as media opacities and various artifacts resulting from blinking or saccadic eye movements. Based on the signal quality acquired by the device, a signal quality score is automatically calculated for each scan. Previous studies highlighted the importance of the image quality as it affects the measurement error which is significantly larger in scans with lower quality scores [110–115]. However, with the advantage of being a non-invasive procedure, the use of OCTA is quickly expanding in the clinical practice. For example, OCTA is being used for early detection of microvascular alterations in diabetic retinopathy, visualizing the boundaries of nonperfusion in vascular occlusion, and following patients with age-related macular degeneration. OCTA is also a promising tool in diagnostics and monitoring of glaucoma.

### Dynamic retinal vessel analysis (DVA)

Dynamic retinal vessel analysis (DVA) allows visualization and direct measurement of neurovascular coupling responses by assessing the change in the retinal arteriolar diameter in response to photoreceptor stimulation. Similar to functional hyperemia in the brain, neurovascular coupling responses in the retina play an important role in matching regional blood flow to the energetic demands of activated neurons through a process in involving astrocyte activation and endothelial release of the vasodilator NO [37, 68]. Pathophysiological conditions that impair neurovascular coupling in the brain are thought to impair neurovascular coupling also in the retina. In DVA, neurovascular coupling in the retina is elicited by flickering light stimulation and the changes in retinal vessel diameter are monitored via a mydriatic fundus camera. Flicker-evoked retinal vascular dilation has good repeatability over a short period of time in healthy subjects [116], as well as those with or at risk for cardiovascular and atherosclerotic disease [117]. As a result, the method has been suggested to be a good candidate to monitor treatment effects in patients with increased cardiovascular disease risk [118], and it has already been used to assess endothelial and microvascular dysfunction in patients with other forms of cardiovascular disease and cardiovascular risk factors, including obesity, hypercholesterolemia, coronary artery disease, and heart failure [104, 117, 119–122]. The utility and sensitivity of DVA for assessing subtle, early stage changes in vascular function is underscored by the finding that DVA can detect microvascular dysfunction in pre-diabetic patients which is worsened as type 2 diabetes develops [123]. This ability to non-invasively assess early aspects of disease pathology is critical for mechanistic studies, and would play an integral part in advancing our understanding of the microvascular complications of CAS. Apart from assessment of the magnitude of arteriolar dilation, the method yields additional functional biomarkers, including indices of the dynamic characteristics of the response [122]. The overall sensitivity, repeatability, and non-invasiveness of DVA make it an exciting translational tool for evaluating microvascular function and neurovascular coupling in patients, and it is not surprising that its use is expanding rapidly.

## Ocular manifestations of carotid artery disease

**Fig. 4:**
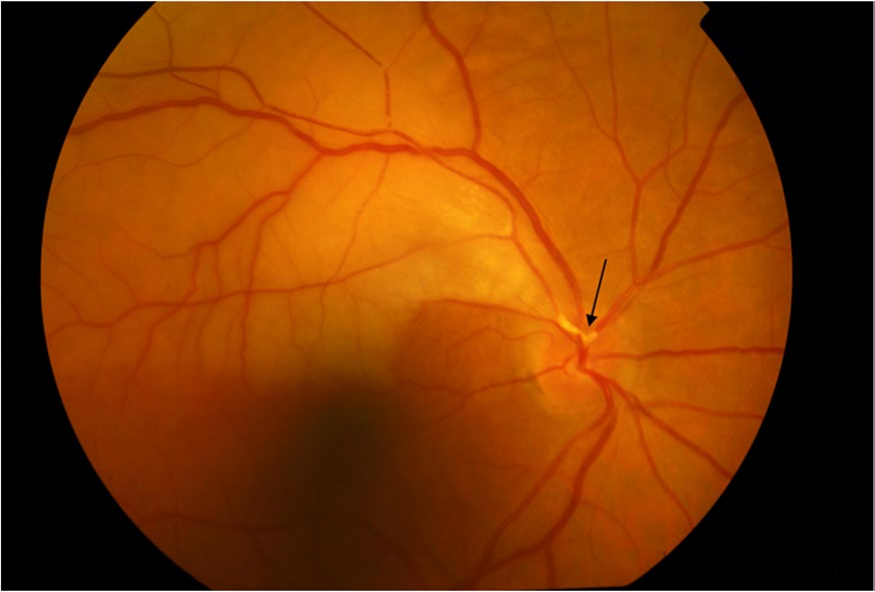
Branch retinal artery occlusion showing retinal whitening in the area of blockage due to a calcified embolus on color fundus photograph (arrow)

**Fig. 5:**
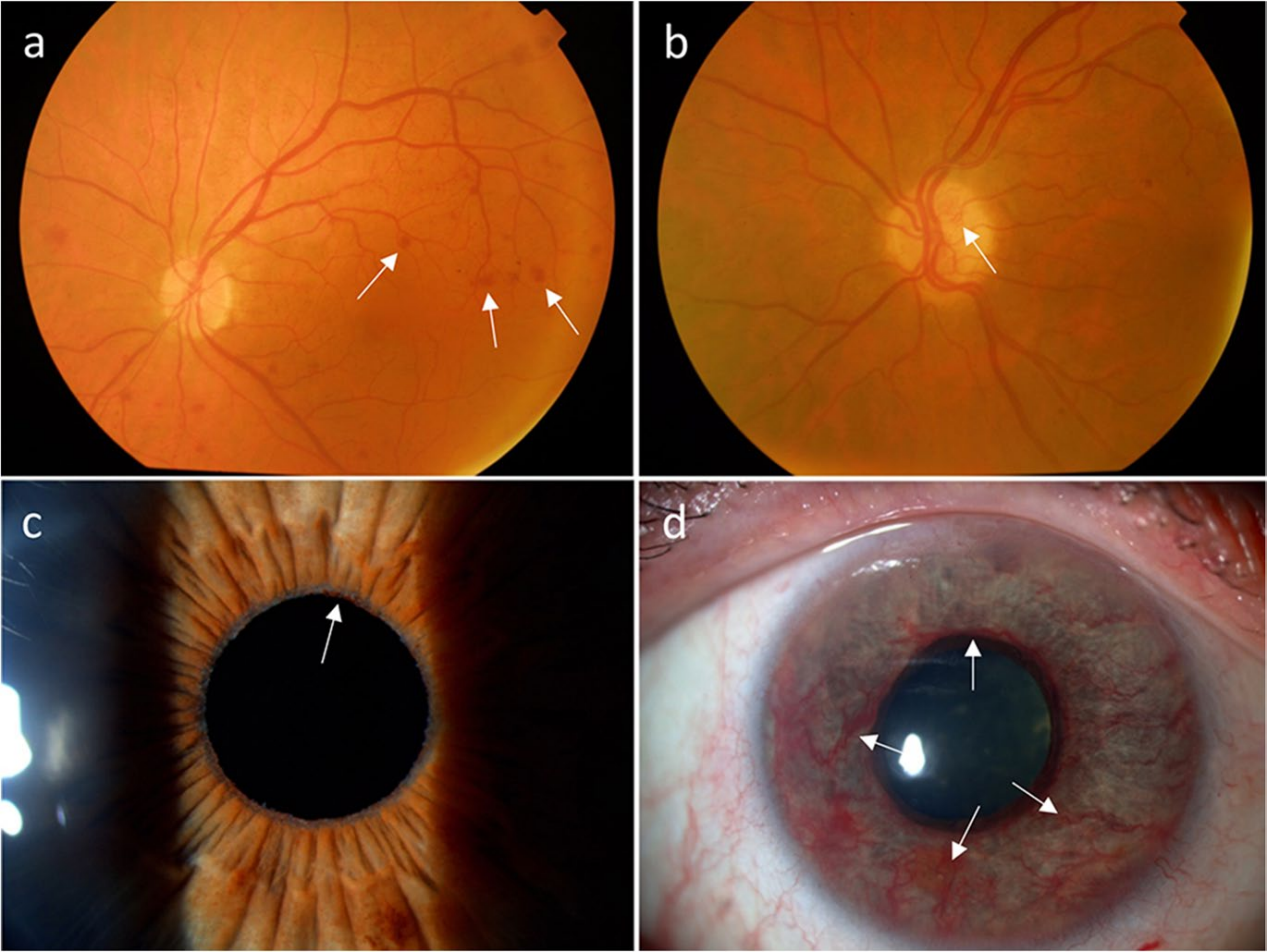
Pathologic changes in the retina (**a**, **b**) and anterior segment of the eye (**c**, **d**) due to ocular ischemic syndrome. Retinal dot bleedings (**a**, arrow) and optic nerve head neovascularization (**b**, arrow) indicates profound retinal ischemia. Chronic ocular ischemia leads to anterior segment neovascularization initially in the pupillary margin (**c**, arrows), and later, on the surface of the iris (**d**, arrows)

The eye receives its blood supply from the ipsilateral internal carotid artery through the ophthalmic artery. CAS can result in severe ophthalmic complications. A temporary decrease in blood flow can cause a painless unilateral transient vision loss termed amaurosis fugax. Amaurosis fugax is a type of TIA, caused by an embolus derived from the ipsilateral atherosclerotic carotid artery which transiently occludes the retinal arteries causing retinal hypoxia [124]. In severe cases of CAS, amaurosis fugax may also develop due to retinal hypoperfusion when the retina is exposed to bright light and its metabolic demands increase beyond what can be met by functional hyperemia [125]. The duration of vision impairment usually varies from seconds to minutes but in rare cases it can last for hours and resolves spontaneously. Embolization of the retinal arteries also can lead to a sudden, painless, but permanent visual loss by causing a retinal stroke. Depending on the size of the emboli, and the location of the stenosis, this can manifest as visual field defects or total vision loss in one eye if the central retinal artery is affected. The results of retinal stroke can be seen in the posterior segment of the eye. In the acute phase, there a whitening of the affected parts of the retina, which is a result of intracellular edema is the most notable change (which later resolves). In around half of the cases, retinal emboli may be seen on funduscopic examination (Fig. 4) [126]. If the condition remains untreated, ocular ischemic syndrome can develop. In these cases, vision loss develops gradually and is accompanied by severe pain. Ocular findings include changes in the anterior segment such as neovascularization in the iris and the iridocorneal angle, opalescence of the aqueous humor, as well as in the posterior segment where narrowed retinal arteries and dilated retinal veins, retinal hemorrhages, microaneurysms or a cherry-red spot in the macula can appear (Fig. 5). Retinal embolization is associated with increased risk of stroke [127–129], illustrating the shared etiologies of vascular pathologies affecting the brain and the retina.

## Effect of carotid artery disease on retinal function and structures

Several studies described functional and morphological changes of ophthalmological parameters in patients with CAS either compared to healthy individuals or between the two eyes of patients with unilateral stenosis. Early studies focused on perfusion and functional changes [130, 131]. In the past few years, several studies have utilized OCT and OCTA as they are rapidly evolving examination methods for evaluating changes in retinal structure and microcirculation even before the manifestation of ocular symptoms related to CAS.

### Functional changes associated with carotid artery disease

Kofoed et al. assessed ophthalmic artery systolic pressure by ocular pneumoplethysmography and performed fluorescein angiography in order to evaluate retinal perfusion and examined retinal function by multifocal electroretinography (mfERG) in patients with asymmetric significant carotid artery stenosis but no clinically manifest eye disease [132]. In the eyes ipsilateral to the highest degree of stenosis, N1 and P1 implicit times (a measure of photoreceptor and bipolar cell response kinetics) were significantly prolonged compared to the contralateral eye. Summed peak N1 and P1 amplitudes (a measure of photoreceptor and bipolar cell function) in the high-stenosis side were also impaired compared to the alternate eye. A correlation between arterial blood pressure and changes in mfERG results was observed on the side with higher degree of CAS [132]. In another study, retinal dark adaptation (an additional measure of photoreceptor function and kinetics) was impaired in CAS patients compared to controls but no significant difference was found between the symptomatic and asymptomatic side [130]. Prolonged or impaired dark adaptation has been associated with other vascular eye diseases such as non-proliferative diabetic retinopathy [133], and in diabetic retinopathy this functional defect is correlated with a significant decrease in retinal perfusion density [134]. Consistent with this, retinal flow density values measured with OCTA are reduced in CAS patients compared to healthy controls [135]. As in the brain, where defects in neurovascular coupling can lead to neuronal dysfunction and cognitive decline, defects in retinal function in CAS patients may be tied to impaired retinal neurovascular coupling. Support for this hypothesis comes from a recent DVA study evaluating CAS patients [136]. Flicker stimulation-evoked retinal venous dilation was decreased significantly in both eyes of CAS patients versus controls [136]. Combined these findings support a link between defects in retinal neuronal function, retinal vascular dysfunction, and CAS.

### Changes in retinal morphology and retinal blood flow

#### Structural changes of the retina associated with CAS

Sayin et al. compared spectral domain optical coherence tomography (SD-OCT) parameters in patients with CAS to a healthy control group and found a significant decrease in macular choroidal thickness which showed no correlation with the degree of the stenosis. They also evaluated the thickness of the retinal nerve fiber layer (RNFL), the macula and the ganglion cell complex (GCC) but found no difference between the two study groups [137]. Another study evaluated the average RNFL thickness and macular thickness in the nine Early Treatment Diabetic Retinopathy Study (ETDRS) areas in CAS patients and found them to be thinner than comparable regions in a control group, however the difference in the RNFL thickness did not attain statistical significance [138]. In a recent study, Wang et al. examined a large number of asymptomatic CAS individuals with SD-OCT and transcranial Doppler and carotid duplex ultrasound to assess the carotid arteries and retinal structure. The study reported a correlation between the presence of a stenosis and the thinning of the RNFL suggesting that such ophthalmological findings may indicate an asymptomatic carotid artery disease [139].

#### Changes in ophthalmic artery blood flow velocity associated with CAS

Studies that have used Doppler sonography to compare blood flow parameters of patients with carotid artery stenosis to healthy individuals found a significant decrease in mean central retinal artery blood velocity and the average systolic velocity of the posterior ciliary artery [131]. In a study comparing the two eyes of patients with CAS, Heßler et al. reported a significant decrease in the peak systolic velocity in the central retinal artery on the CAS side, although they found no difference in morphological and functional changes in the retina (such as RNFL thickness, total macular volume, optic nerve head volume and visual acuity) [140]. In other studies, the effect of carotid endarterectomy was evaluated. In patients with ocular ischemic syndrome due to ipsilateral carotid artery stenosis, color Doppler ultrasound of the ophthalmic artery was performed before and after surgical treatment. Multiple studies found an improvement in ocular blood flow postoperatively and found that endarterectomy was effective in improving or preventing the progression of ocular ischemia caused by CAS [141, 142].

#### Effects of CAS and its surgical treatment (carotid endarterectomy) on the retina

The effect of carotid endarterectomy on ocular manifestations of CAS has been widely studied in the recent years. Yan et al. observed an improvement in both subjective and objective visual function (such as visual acuity, static and kinetic visual fields, visually evoked potentials and electroretinographic parameters) in patients after surgical treatment [143]. However, no changes were detected regarding the RNFL thickness [143]. In contrast, Guclu et al. reported a statistically significant decrease in peripapillary RNFL thickness in the superior quadrant following surgery [144]. Another study evaluated the effects of endarterectomy on retinal and choroidal thickness using enhanced depth imaging OCT (EDI-OCT) [145]. Retinal thickness was not altered in CAS patients vs. controls or in pre- vs. post-endarterectomy CAS patients. In contrast, choroidal thickness was reduced in CAS patients, and this impairment was corrected (i.e., a significant increase in choroidal thickness) after endarterectomy. Interestingly, this increase in post-surgical choroidal thickness was noted only in cases where the extent of the stenosis was 50–70% but not in patients with a higher degree of stenosis [145].

These post-surgical benefits to retinal structure and function benefits may not be accompanied by improvements in retinal neurovascular coupling. For example, the decreased flicker stimulation-evoked retinal venous dilation in CAS patients discussed earlier was not improved after carotid endarterectomy [136]. These results suggest that CAS-associated microvascular dysfunction may not be resolved by restoring the luminal diameter of the carotid artery. However, this area of research is still evolving and care must be taken with interpretation of findings. DVA measurements assess only the function of the retinal vasculature, while retinal function relies both on the choroidal vasculature (which has distinct hemodynamic parameters from the retinal vessels) and the retinal vasculature. However, it appears that DVA is a sensitive method to detect microvascular dysfunction caused by systemic risk factors that contribute to CAS.

**Fig. 6:**
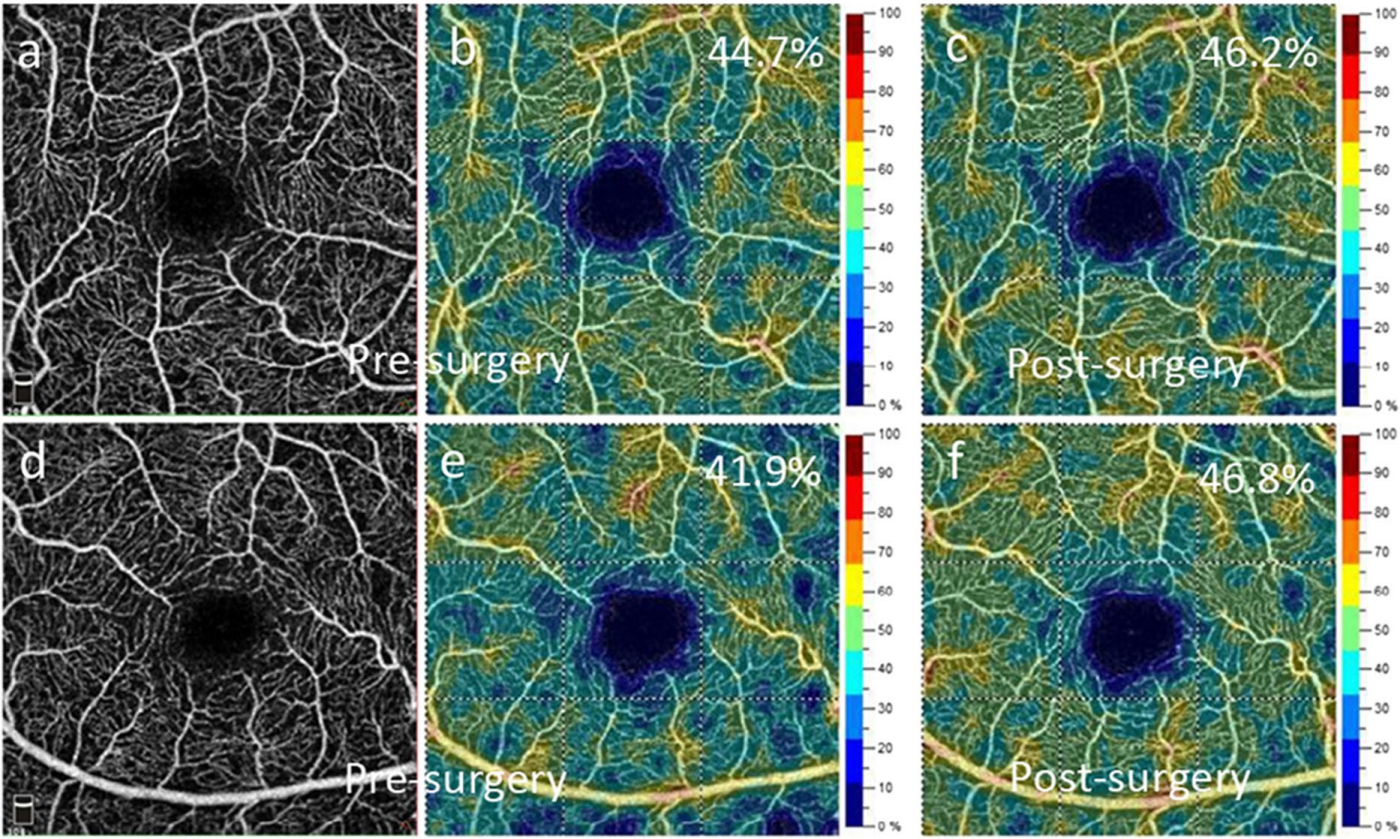
Retinal blood flow imaged using OCTA in the right (**a**–**c**) and the left eye (**d**–**f**) of a patient with significant left carotid stenosis before surgery (**a**, **b**, **d**, **e**) and after carotid endarterectomy (**c**, **f**). The AngioAnalytics software of the OptoVue AngioVue system provides en face angiograms (**a**, **d**) and color coded images for vascular density values in the 3 × 3 mm macular area (**b**, **c**, **e**, **f**)

The surgical benefits of endarterectomy on retinal flow density are similarly complicated. One group demonstrated that the reduced retinal flow density in CAS patients was improved post-surgery in the radial peripapillary capillary network of the optic nerve head but not in the superficial or deep layers of the macular area [135]. Another study evaluated the retinal microcirculation after carotid angioplasty with stenting in patients with severe CAS [146]. A significant increase in vessel density was detected in the macular deep vessel complex both on the ipsilateral and contralateral eyes after surgery. In the contralateral eye the vessel density in the superficial layer improved as well [146]. In the clinical setting we also observe similar patterns. Figure 6 shows impaired retinal blood flow on the ipsilateral side of patient with CAS, and an improvement in vascular density values in both eyes of a patient with left carotid stenosis after endarterectomy. Taken together these data support the idea that surgical treatment of CAS may provide bilateral improvement in retinal vascular perfusion, and that OCTA is a useful method to assess alterations in retinal blood flow due to CAS.

Table 1 summarizes the most important cerebral and retinal structural and vascular biomarkers associated with carotid artery disease that can be used to assess cerebral blood flow impairment.

**Table 1:** Cerebral and retinal structural and vascular biomarkers associated with carotid artery disease

Structural and vascular biomarkers	Imaging technique	Quantification possible?	Reason in favor of use	Principal limitation
Carotid morphology	CTA MRI	NASCET criteria	Anatomical structures variance assessment Plaque analysis	Over estimation of calcification Under estimation of calcification
Carotid blood flow velocity	DUS	PVS > 125 cm/s EDV > 140 cm/s	Real-time hemodynamical information	Investigator dependent
Cerebral morphology	CTA or MRI	No	Subacute and chronic ischemic lesion detection Anatomical structures variance assessment	Image resolution limits the visualization of smaller vessel
Cerebral blood flow velocity	TCD	No	Measurement the cerebral reserve capacity	20% of patients lack of TCD window
Retinal neuronal changes				
Macular RNFL thinning	OCT	Yes	Quantification of retinal neural damage	Lack of information on retinal blood flow
Peripapillar RNFL thinning		Yes		
GCL-IPL thinning		Yes		
Retinal vascular changes				
VD decrease	OCTA	Yes	Quantification of retinal capillary blood-flow	Not part of routine clinical practice
FAZ enlargement		Yes		

*DUS* duplex ultrasound, *PVS* peak systolic velocity, *EDV* end diastolic velocity, *CTA* computed tomography angiography, *MRI* magnetic resonance imaging, *TCD* transcranial Doppler, *OCT* optical coherence tomography, *OCTA* optical coherence tomography angiography, *RNFL* retinal nerve fiber layer, *GCL-ICL* ganglion cell layer-inner plexiform layer, *VD* vessel density, *FAZ* fovea avascular zone

## Concluding perspectives

CAS and CAS-related cognitive impairment are important health concerns in the aging societies of the Western world. Early detection of microvascular contributions to VCI and assessment of the efficiency of therapeutic approaches are key to improved patient outcomes. The retina and the brain have striking pathophysiological similarities, and as both the retina and the brain are downstream from the carotid artery, the retina may provide a window to enable non-invasive assessment of the severity of CAS complications in the CNS distal to the lesion. Likewise, due to the anatomical and physiological similarities between the brain and the retina, evaluating structural and vascular changes in the latter can bring us to a better understanding of the effect of CAS on the central nervous system, and in particular, the role of the microcirculation in CAS-associated cognitive decline. Numerous studies have reported alterations in the retinal structure and vasculature associated with CAS that mirror many changes that occur in the brain. Recent advancements in retinal imaging make fast and non-invasive visualization of the retinal structure and blood flow possible. Among powerful new methods, OCTA is an especially promising tool for detecting early changes related to CAS and assessing the effect of surgical treatment on the changes in the CNS microcirculation. For research purposes, using these ophthalmological methods provides a unique opportunity to image CAS related neurodegenerative processes longitudinally in vivo. Furthermore, the emerging ability to use DVA and OCTA imaging in animal models provides an opportunity to evaluate cellular and molecular mechanisms underlying microvascular complications of diseases such as CAS and correlate findings with similar outcomes in patients.
